# Final Report of the Committee of the Philadelphia Medical Society, on the Construction of Instruments, and Their Mode of Action, in the Radical Cure of Hernia, &c.

**Published:** 1838-10

**Authors:** 


### Art. V.

1. *Final Report of the Committee of the Philadelphia Medical Society, on the Construction of Instruments, and their mode of action, in the radical Cure of Hernia, &c.* By Heber Chase, m.d.—*Philadelphia*, 1837. 8vo. pp. 243.
2. *De la Cure Radicale des Hernies.* Par M. le Docteur Belmas, Ancien Chef des travaux Anatomiques de la Faculté de Médecine de Strasbourg. (*Revue Médicale francaise et étrangère.* No. 2 and 3. —*Février et Mars*, 1838.)
3. *Uber radicalen Heilung reponibler Brüche.* Von Dr. Ph. Finck, Grossherzogl: Badischem Militärarzte. *Mit Zwei Kupfertafeln.*—*Freiburg*, 1837. 8vo. pp. 48.

That mode of treating reducible hernia which has for its object a radical cure of the complaint, and not a mere palliation of the inconveniences attending it, has engaged the attention of surgeons in all countries, and at all periods. Within the last few years, the subject seems to have received a fresh impulse, both on the Continent and in America, where considerable talent, combined with much patient ingenuity, appears to have been bestowed on this important branch of surgery. On this account we purpose, in this article, to lay before our readers a concise summary of what has hitherto been attempted and accomplished with regard to the radical cure of hernia, and to institute a comparison between the different methods proposed and executed by the contemporary surgeons of France, Germany, and America.

As the inguinal or scrotal hernia is by far the most common, the most unmanageable, and entails the greatest degree of evil and suffering on those afflicted with it, and as it has, consequently, received the greatest share of attention from surgeons, our remarks and enquiries will chiefly refer to this particular species of rupture.

Almost every variety of hernia may be described as the escape of part of the contents of the abdomen through one of those openings which have a normal existence, and which form communications between the interior and exterior of the cavity, for the transmission of vessels, nerves, or other structures. Thus a hernia can hardly be said to establish a new opening, but rather consists in the dilatation, accompanied by alteration as to form and direction, of one which existed previously in the healthy state. This view of the subject is strikingly exemplified in the formation of a common inguinal hernia. The animal economy renders a communication necessary by means of the spermatic cord, between the testes and the abdominal cavity, and nature, foreseeing the risk which this necessity involves, has, as it were, exerted all her ingenuity in rendering the communication of such a kind as shall allow the transmission of vessels, nerves, absorbents, and ducts, without compromising the integrity of the abdominal walls through which they have to pass. Hence the oblique and lengthened course of the cord as it traverses the walls of the belly; hence the adaptation of fasciæ and tendinous structures around the different openings, maintaining the security of the internal and external abdominal rings. A hernia in its descent slowly but surely undoes the work of nature, destroying step by step the barriers purposely opposed to such an accident: the inguinal canal becomes gradually dilated, and as the parts which surrounded the cord yield and expand before the superincumbent pressure, so does the obliquity and length of the passage disappear, until a nearly straight and direct communication becomes established between the abdomen and the scrotum; leaving scarcely a trace of what, in the anatomical language of the schools, are commonly but erroneously denominated "the parts of hernia." It is a dilatation rather than a rupture; but at all events there is a breach, a deficiency of the abdominal parietes; and a radical cure can only be brought about by such means as will lead to the filling up or closure of the abnormal opening.

The rationale of all methods adopted to promote a radical cure may be resolved into two principles, 1st. The prevention of protrusion until nature has had sufficient time to remodel the parts, to rebuild the fabric which the hernia has destroyed; in other words, to fill up the opening and restore the integrity of the abdominal walls. 2d. The establishment by artificial means of so much inflammation either within or around the neck of the hernial sac, or in both these situations, as shall produce adhesion and agglutination between the separated parts, and thus obliterate the opening which allowed the descent of the protruded viscera.

I. The first of these principles, or that of allowing nature to contract and close the opening, is of course only to be carried into effect by the application and adjustment of trusses. Its success depends entirely on the skilful construction and the accurate adjustment of the mechanical contrivance, which, as a work of art, belongs as much to the artificer as to the surgeon. We have no hesitation in affirming that the only way in which a truss can be rendered available in producing a radical cure, is by its preventing all protrusion or even tendency to protrusion. Therein consists the excellence of its mechanism and its adjustment. In its application, the nearer it can be brought to resemble the natural walls of the abdomen, the more effectual it will be. It should just afford as much support to the abdominal walls as will compensate for the inability to resistance which has led to the complaint; and it should just occupy or oppose itself to the breach which the previous descent of the hernia has left. As regards the promotion of a radical cure, we believe that a truss can do no more than to prevent the process of nature, in contracting the opening and restoring the walls to a normal condition, from being interrupted and thwarted by the occasional recurrence of a fresh protrusion: and where the resources of nature are inadequate to repair the breach, we are inclined to deny the efficacy of a truss in stimulating or assisting her to effect the object, otherwise than in the manner just mentioned. In such cases the truss becomes a palliative not a curative remedy. Trusses have indeed been constructed for the express purpose of producing irritation on the surface against which they are applied, and of exciting so much deep-seated inflammation as to condense the cellular tissue, fascia, and tendon about the hernial orifice, into one common mass by adhesion, thus carrying into effect the second of the two principles upon which the rationale of a permanent cure must be founded. These instruments have never stood the test of experience and fair investigation. Their application and use are in many instances so intolerable to the patient, as to render the remedy far worse than the disease; and, if persevered in, their pressure tends rather to produce a gradual absorption of the abdominal walls than that thickening and consequent increase of solidity and strength which they were intended to promote. The report of the Philadelphian Committee condemns, in decided terms, the trusses of Messrs. Stanger and Wood, which were constructed on this principle and furnished with blocks of wood, lead, and iron, exerting a strong pressure with the view of exciting inflammation and adhesion. The committee represent them as unnecessarily severe for the purpose of preventing protrusion, and utterly useless if not mischievous as far as regards any ulterior object. Returning, then, to the axiom which we just laid down, viz. that a truss is only useful in producing a radical cure inasmuch as it allows nature an undisturbed opportunity of healing the breach, it becomes a matter of the deepest importance, as regards the diagnosis and treatment of hernia, to ascertain the positive efficacy that may be anticipated from the use of a truss under the different forms and degrees of hernia; not merely its efficacy to retain the bowel, but to accomplish a cure within a reasonable period of time.

If we are to place implicit credence in the assertions and the alledged experience of the different inventors and improvers of mechanical contrivances for support and retention, we must believe that the truss is allsufficient to prevent protrusion, and that the vis medicatrix naturæ is in every instance competent to perform its part in the removal of the complaint: the genius of the mechanic has triumphed, and precludes the necessity of farther interference on the part of the surgeon.

We must refer our readers to a former number of this journal, (Vol. V. p. 300,) and to the work which heads the present article for a description of the trusses invented by Dr. Chase, and the success which has attended their use. He seems to have brought the instrument to a perfection which it had never previously attained, and the Philadelphian Committee, whose testimony is certainly entitled to our confidence and respect, report in the highest terms, of its efficacy in producing a radical cure, not only in ordinary cases, but in many which held out but little prospect of so fortunate a result. We must, however, observe that the report of the committee must necessarily have been confined to those cases which were brought under their own observation, and these, in all probability, by no means included all the individuals who had been subjected to the apparatus recommended by Dr. Chase, although he certainly seems to have given every opportunity of submitting his mode of treatment to a fair and impartial trial. In the report referred to in our ninth Number, Dr. Chase's instruments are figured, their mode of application minutely described, and the general results derived from their use exposed at length; otherwise we should have thought it necessary to lay before our readers, in this place, such an account of them as might enable the surgeon fully to comprehend their mechanism both of construction and application, in order that he might himself test their efficacy. We must therefore request our readers, before going further, to peruse the document, which he will find well worthy his best attention. We are far from thinking that the mechanical contrivances for preventing protrusion have in this country received all the improvements of which they are capable. Too much has generally been left to the instrument-maker and too little has been suggested by the surgeon; but, at the same time, we feel convinced that cases of hernia will be constantly met with, where no expectation of a radical cure through the application of a truss, can possibly be entertained; and where the surgeon feels himself called upon to tell his patient that he must make up his mind to wear the instrument as a palliative for the remainder of his life.

II. From the time of Celsus to the present period, attempts have been constantly renewed to produce obliteration of the hernial opening, either by attacking the dilated inguinal canal from without, through the agency of severe pressure, escarotics and actual cauteries applied to the surface of the skin; or else by the bolder method of at once penetrating to the seat of mischief and endeavouring, by various methods, to stimulate the neck of the peritoneal sac itself, and its immediate investments, to inflammation and adhesion. We have already noticed the futility and the mischievous effect of high-pressure trusses: they have been employed at different times by Paré, Petit, Richter, Langenbeck, Arnaud, and a long list of less distinguished names, either simply or combined with irritating applications; and while they appear, in many instances, to have failed in effecting a cure, the remedy has occasionally involved the patient in fresh disasters, not only by subjecting him to a long and tedious confinement, but by giving rise to extensive sloughing of the external parts, which in more than one case terminated fatally. In fact, the skin does not appear capable of transmitting sufficient inflammation to the parts beneath without itself becoming destroyed in the process, and all external remedies appear to have failed in effecting the desired object.

Our ancestors, however, were not deficient in resources for striking at once at the root of the evil and applying their means to the neck of the sac and the region of the inguinal canal. Many were the contrivances put into practice, some of which savour strongly of the adventurous and reckless spirit which animated the older surgeons: thus, the neck of the sac was firmly tied up by means of a ligature passed around it immediately below the external ring, and frequently including the spermatic cord as well as the integuments; or else it was sewn up by a succession of stitches with an equal disregard to the neighbouring structures. Such operations, by which the loss of the testis was compromised and the life of the patient endangered, were nevertheless enobled by the names of "Golden Incision," "Royal Suture," &c. The latter of these methods, including a few additions, as practised by the surgeons at Constantinople, is so graphically described in a French history of the Ottoman Empire, and affords so striking an example of the heroic practice of the times, that we shall be excused for transcribing the original passage. The author after alluding to the means employed for the cure of rupture by the practitioners of the sublime Porte proceeds to say—"Ils l'entreprennent sur toutes sortes de gens; durant mon séjour à Constantinople, j'en fis l'épreuve sur mon secretaire. Ils le lièrent sur une forte planche, ils ouvrirent le sac, réduisirent les parties sorties, puis firent une suture en enlevant avec un rasoir toute la portion de peritoine qui se trouvait au-devant d'elle. Après quoi ils couvrirent la plaie avec de la graisse de porc, y mirent le feu avec un fer rouge, et arrosèrent ensuite les parties avec des blancs d'œufs. Le patient ne donnait plus aucune signe de vie, et quand il revint à lui ce fut pour se plaindre de violentes douleurs dans le ventre, &c." We are not informed whether the secretary was fortunate enough to survive this treatment, or whether the inflammation produced was effectual in closing the hernial opening.

The operation of surrounding the neck of the sac by a ligature to the careful exclusion of the adjacent parts, was likewise practised and recommended by Paré, Heister, and Schmucker, also by Désault and Sharpe; but the method was finally abandoned in consequence of the pain and danger attendant upon it and its frequent inefficacy in producing a radical cure. Several other contrivances proposed and practised with a view of obliterating the communication between the hernial sac and the peritoneal cavity of the abdomen shared the same fate: these consisted in scarifying or applying caustic to the neck of the sac, injecting it as in hydrocele, &c.

A more recent attempt on the same principle, and with somewhat better success, has been made by M. Bonnet, chief surgeon of the Hôtel Dieu of Lyons, which we think of sufficient importance to notice more at length. Several cases treated in this manner have been published very recently in a French journal.

In the first case, operated on by M. Bonnet, he passed eight pins through the hernial sac, near the inguinal ring. These were maintained in their situation by a cushion and a bandage. On removing the bandage, three days afterwards, the pins had all escaped. The object of

M. Bonnet was next to pass the pins completely through the root of the scrotum, and to twist their points so that they could neither pass in nor out. In order to prevent their sinking into the inflamed skin, and to effect a sufficient degree of pressure upon the parts situated between the extremities of the pins, he enlarged them by means of a piece of cork. The hernia being reduced, the root of the scrotum was seized as close as possible to the abdominal ring, and the cord placed at its external side, in a circle formed by the left thumb and index finger. The points of these fingers being forcibly pressed together, he passed a pin in front of their nails, behind the coverings of the hernia, and near the suspensory ligament of the penis. Having inserted it, until its head rested upon the skin, and its point escaped in front, he passed this point into a piece of cork, which he pressed upon the point until the parts between the cork and the head of the pin were somewhat compressed. The point was then fixed by twisting it with a pair of pincers. The first pin being thus fixed, he carried the cord between it and the points of the left thumb and index finger. He then, following the ends of his fingers, passed a second pin parallel to the former, situated from six to seven lines farther outwards. At the same distance was then placed a third and a fourth. The pins were removed from the sixth to the twelfth day, and, as soon as the sensibility of the parts permitted, a compressing bandage was applied. As this process was not sufficient, a second row of pins was inserted, so as to multiply the points of adhesion, and these pins were allowed to remain in until the ulceration of the skin was complete, and the compression was exercised upon the cellular tissue itself. But, notwithstanding this improvement, M. Bonnet effected but a temporary and apparent cure on the only old person upon whom he operated. He failed in three operations upon adults for very large herniæ, which escaped from the inguinal canal, and which had become direct and large enough to allow of the simultaneous introduction of several fingers. He had complete success however on three adults, whose hernia were as large as the fist, and in whom, the inguinal canal, still oblique, allowed the introduction of but one finger. And a similar success attended the operation on a child of ten years old, where the hernia descended to some inches above the knee, after he had taken continued exercise.*

But among the modern propositions and plans for the radical cure of hernia by the obliteration of the passage, conceived in a spirit more in accordance with the advanced state of our science, those recently introduced by MM. Belmas and Gerdy deserve a more circumstantial notice and fuller consideration. Each of these surgeons has chosen a different road to the attainment of his object. The principle carried out in the operation of Dr. Belmas, is the closure of the neck of the sac by exciting adhesive inflammation on the serous surface itself; while Mr. Gerdy has followed up the method of closing the dilated canal and rings through which the sac had descended from the abdomen. The one addresses his remedial measures to the peritoneal communication between the scrotum and the belly, the other seeks to supply by artificial means the deficiency in the abdominal walls which has led to the abnormal protrusion. The first is a modification of the principle which occupied the attention and

* Bull. gen. de Therapeutique. T. x. 9e liv. Mai, 1836.

exercised the ingenuity of so many of the older surgeons: the latter presents altogether a new feature as regards the application of surgical means for the cure of reducible hernia. We shall give an abridged account of the two methods, and endeavour to compare them with each other, both as relates to the operations themselves, and the results which may reasonably be anticipated from them. We ought to state here that the small work of Dr. Finck contains a brief and accurate account of these methods, and also short references to those proposed by former surgeons: it is a respectable compilation, and does not affect to supply any original information.

III. With a view of making himself acquainted with the safest and most certain mode of exciting a local adhesive inflammation between the opposed surfaces of a serous membrane, Dr. Belmas instituted a series of experiments on animals, and finally ascertained, that when a small bladder of gold-beater's skin, inflated with air, was introduced into the peritoneal cavity of a dog, the spot to which the foreign body had applied itself was indicated, after a lapse of three months, by the presence of a globular adhesion ("adherence globuleuse") a sort of fibrous nodule, ("noyau fibreux"), while the general peritoneal cavity remained free from any other trace of inflammation and adhesion. The gold beater's skin itself had disappeared, that is to say, it had either been absorbed, or else become so completely identified with the lymph effused, as no longer to be distinguishable.* Dr. Belmas having now, as he considered, discovered an animal substance, the presence of which was sufficient to produce a mild form of adhesive inflammation, purely local in its character, between the walls of a serous cavity, and which would be spontaneously removed by absorption after accomplishing the desired object, proceeded to make trial of its efficiency in procuring the obliteration of a hernial sac. Thirty dogs afflicted with rupture were selected for experimental operation. Inflammation was excited in the hernial sac, more especially about its neck, by the introduction of gold-beater's skin. In the majority of cases obliteration of the sac was obtained; in some it merely became diminished in size, but in no instance was the operation followed by serious mischief. From this, Dr. Belmas felt himself justified in applying his principle to the human subject. The operation of conveying the gold-beater's skin into the mouth of the sac was commenced by means of a trochar, which entering the lower part of the sac was carried upwards until it had passed the neck, and then brought out through the integuments just above the external ring. A very complicated apparatus of stilets, canulas, and stop-cocks, contained one within the other and individually removable during the manipulations, was then put into motion. The machine by which a fragment of gold-beater's skin was deposited and left in the sac is minutely detailed, but, as little idea of its construction can be conveyed in words, we shall pass it over, more especially as its use was soon abandoned for another contrivance. Indeed, the severity of the operation and some rather untoward results, subsequently induced Dr. B. to adopt the method of merely puncturing the lower part of the sac with a trochar and canula, and then introducing through the latter one or more portions of the gold-beater's skin supported on sticks of dried and hardened gelatin.

* See a further account of these experiments in our last Number, p. 232.—Eds.

These were pushed up into the neck of the sac and left there. The gelatin soon dissolved, and left the membrane which had surrounded it, in contact with the serous surfaces. To accomplish this object also, a peculiar apparatus was used, the mechanism of which we have in vain attempted to understand, but we are assured that if complicated in structure it is simple in its action. A bandage, or properly prepared truss, must be worn for a certain period after the operation, or until the adhesive process has become confirmed and solid. The result of ten operations is thus stated:—"Two out of the number left the neighbourhood after the operation but have represented themselves as cured. In three other cases the success was placed beyond a doubt. In three more an obliteration was obtained of the neck of the sac, but the subsequent necessary compression at the ring having been abandoned too early, a fresh protrusion of the obliterated point took place: the bandage has been reapplied, and the return of the ring to its normal condition is already such that we anticipate a decided cure. The two remaining individuals were obliged to lay aside their bandages at an early period in consequence of the inconvenience they produced, and in them the herniæ have reappeared: they are anxious to undergo a fresh operation." [*Revue Med. Mars*, 1838. p. 412.]

We conceive that the table of results offers the best commentary on the method adopted by Dr. Belmas for the radical cure of hernia. In our opinion the operation is open to the following objections, which certainly have not been satisfactorily combated by the author in his treatise on the subject.

1. By Dr. Belmas' own showing the operation is by no means easy or simple, requiring considerable practice in the use of a rather complicated apparatus, together with great tact and delicacy of manipulation.

2. The means employed seem occasionally to be inadequate for the accomplishment of the object desired, viz. the sealing up of the neck of the sac, or, at all events, to require the application of pressure for so long a period, as may render it doubtful whether the cure ought to be ascribed to the operation or the continued use of the truss.

3. The success of the operation is confined to the sac itself, and merely cuts off its communication with the peritoneal cavity without diminishing the size of the opening in the abdominal walls through which the hernia descended: consequently, we find that in three instances a subsequent protrusion took place, the neck of the sac being forced down by the pressure of the viscera. This very circumstance must render the operation less efficacious when we have to deal with old voluminous wide-mouthed ruptures, which of all others are the very cases considered to be incurable under the ordinary treatment by means of trusses. Future experience will however afford the best test of the utility of Dr. Belmas' plan, and the trials have certainly at present been too few to warrant us in passing a decided judgment on the question.

IV. The plan adopted by M. Gerdy is, as we have stated, founded on an entirely different principle. He practises a very summary but at the same time effectual method of closing the abdominal opening, by carrying a fold of the scrotal integuments upwards into the external ring, and securing it in that situation by one or more sutures. In fact, by establishing a sort of artificial hernia in an inverse direction, (that is from without to within, or from the scrotum towards the abdominal cavity,) he seeks to fill up the dilated inguinal canal, and thus oppose the future descent of the viscera into the original hernial sac. The principle which has guided him was probably suggested by the result of two cases recorded by Arnaud, wherein, during the reduction of an inguinal hernia, a fold of skin was carried up and drawn into the ring: adhesion took place and the cure was permanent. The operation of M. Gerdy is simple, the only instrument required being a curved needle fixed in a handle and pierced with an eye at a short distance from its point. The patient is placed in the horizontal position, and after the surgeon has ascertained that the hernial sac is empty, and has carefully examined its relative situation and connexions with respect to the spermatic cord and the inguinal canal, he passes the fore finger of his left hand upwards through the external ring, carrying of course before it the skin of the scrotum. A blind pouch of integument resembling the finger of a glove is thus introduced into the inguinal canal, anterior both to the cord and to the neck of the hernial sac. The point of the needle, armed with a ligature, is then guided along the finger to the bottom of the pouch, and by depressing the handle is brought out through the anterior walls of the inguinal canal. One end of the ligature is then laid hold of by an assistant; the point of the needle is withdrawn into the pouch, and a second similar perforation is effected, but at a few lines distance from the previous puncture. In this manner both ends of the ligature are carried from the inside of the pouch, or invagination as it is called, through the inverted skin, through the tendon of the external oblique, the superficial fascia and common integument on to the inguinal region, where they are tied together over a piece of bougie. One or more of these stitches are made according to the size of the opening, and the invaginated fold of scrotum is thus retained in its new situation. Caustic ammonia is then freely applied to the internal surface of the pouch formed by the invaginated scrotum, and the operation is concluded by covering the parts with a slight compress spread with common cerate. About the third day a copious suppuration is established, and at this period the sutures may be removed unless circumstances should warrant their further retention. Nature is then allowed to complete the process which the surgeon has commenced. Adhesion gradually takes place between the fold of invaginated integument and the sides of the inguinal canal into which it has been received, while at the same time the pouch itself becomes obliterated by the process of granulation set up by the caustic. Within the fifteenth or twentieth day the suppuration ceases, and the dilated inguinal canal will then be found completely blocked up by a strong thick plug, the presence of which is indicated by an external projection, and which may be distinctly felt beneath the skin and tendon. This gradually disappears, until no trace of the operation remains except a trifling scar at the entrance of the pouch, and a slight elevation and shortening of the scrotum on that side. The patient should be kept on his back for a month after the operation, and his shoulders and pelvis should at first be somewhat raised for the purpose of relaxing the walls of the belly. The scrotum should also be supported. The patient's diet should be low, and everything that tends to bring the abdominal muscles into action, as coughing, sneezing, difficult evacuation of the bowels, &c. carefully avoided. The progress of inflammation ought to be watched, and the usual antiphlogistic remedies adopted, if it evinces a disposition to exceed the proper degree of intensity or to spread beyond the limits prescribed. A truss should be worn for about three months after the patient has ceased to observe the recumbent posture.

In large old inguinal herniæ where the peritoneal sac is closely connected to the integuments of the scrotum and to the circumference of the ring, there would probably exist some danger of its becoming folded, carried up before the finger during the process of invagination, and subsequently pierced by the needle; this, in fact, has occurred in the practice of Messrs. Velpeau and Gerdy, but does not seem to have been followed by any untoward consequences. The risk of wounding the cord, the epigastric artery, or the abdominal peritoneum itself has been urged as an objection to the operation, but we conceive that such accidents could hardly occur under the hands of a cautious and skilful surgeon.

M. Gerdy has operated on thirty individuals, all of whom were cured, temporarily at least, with the exception of one, who was attacked with pleuritis under which he sunk. We are not, however, furnished with any history of the cases extending beyond the period of their return to their ordinary avocations; and, in all probability, sufficient time has hardly yet elapsed to test the ultimate efficacy of the plan in producing a radical and permanent cure.

We think that the operation of M. Gerdy has many advantages over that of Dr. Belmas. In the first place, it is simple and easy in its execution, much more speedy and certain in its effect, and strikes at once at the root of the disease by plugging up the dilated inguinal canal. It is more calculated to overcome the difficulties presented by a large wide-mouthed hernia, as the number of sutures employed to retain the invaginated fold of integument can always be proportioned to the size of the opening. It likewise involves parts which do not readily resent the injury offered to them; and by leaving intact and undisturbed the serous cavity of the abdomen, offers an immunity against subsequent peritoneal inflammation which no other method can boast of. How far the neck of the hernial sac itself is rendered impermeable through this operation, can only be ascertained by experience and the evidence afforded by post-mortem examination. There can be no doubt that the mouth of the sac becomes narrowed, and, in many cases, may be completely obliterated by the pressure or the extension of inflammation excited in the adjacent parts; but so desirable a result is not necessarily inferred by the principle of the operation; and, should a communication still remain between the original sac and the cavity of the belly, a return of the hernia may possibly take place at some period after the use of the truss has been altogether abandoned. M. Gerdy does not appear to have confined his practice to those cases which held out no other prospect of relief, but to have operated indiscriminately for all kinds and conditions of ruptures. This we infer from the recommendation which he gives to return the sac as well as its contents previously to commencing the operation; and from the directions, that when the ring is too small to admit the finger, the invagination is to be accomplished by means of a curved hollow staff, through which the needle may be introduced. Of course this advice can only relate to the treatment of small or recent hernia, which we should consider remediable by the use of a truss.

The limits of this article will not allow us to extend our remarks; but we have been induced to direct our attention to this subject, not only because we are convinced that there are many cases of hernia which are incurable by the common palliative mode of treatment however judiciously applied; but also, because a speedier and more certain cure than can ever be accomplished by the use of trusses, would be an inestimable boon to a very numerous class of individuals. We allude more especially to the lower orders, to the working people who are the principal sufferers from rupture, who may be said to earn the complaint by their bodily labour, by the sweat of their brow, and in whom a continued necessity for renewed exertion almost precludes the possibility of rendering available those gentler means of cure which may be successfully adopted among the higher classes of society.

We think that the radical cure of hernia has in this country hardly received that share of attention which so important a subject deserves. We would ask our professional brethren to remember the many instances they must have witnessed, where the patient has sunk after the operation for strangulated hernia, often performed under the most disadvantageous circumstances; and we would then remind them that nine times out of ten the strangulation has supervened on an old rupture, which, through carelessness, ignorance, or poverty, has been suffered to exist from year to year, either totally disregarded or at best imperfectly supported. We conceive that, in the treatment of reducible hernia, the attention of the surgeon should be more strongly directed than it is at present towards the accomplishment of a radical cure. His knowledge and his experience will alone enable him to decide upon the most efficacious means for attaining this object and placing his patient beyond the reach of accident, whether by the use of trusses or by operation. It is not our intention to enter into the circumstances and symptoms which will guide him in the choice of the former or the latter remedy; but the size of the rupture, the period of time it has existed, the nature of its communication with the abdominal cavity, the age of the patient, his temperament, his condition in life, the occupation he is obliged to follow,—must all be taken into consideration as influencing the decision. In some instances it is probable that the inclinations of the individual himself may settle the question, leading him to prefer an operation as the speediest method of restoring him to a sound condition.

We have given a free but we hope an impartial opinion on the different operations for the cure of hernia, which have formed the principal subject of this article. They have yet to stand the test of time and experience before their respective merits can be definitively settled; but we are sure that the profession, and the public in general, will owe a tribute of gratitude to that surgeon, who shall succeed in rescuing a numerous class of his fellow creatures from an affliction, which, like the sword of Damocles, serves to embitter every enjoyment, and places their lives in continual jeopardy.

